# Clinical Predictors of Severe Exacerbations in Pediatric Patients With Recurrent Wheezing

**DOI:** 10.7759/cureus.52667

**Published:** 2024-01-21

**Authors:** Margarida Serôdio, Catarina Albuquerque, Marta Figueiredo, Joana Moscoso, João Serôdio, Rita Barreira, Rita Monteiro, Maria João Leiria

**Affiliations:** 1 Pediatrics, Hospital de São Francisco Xavier, Centro Hospitalar de Lisboa Ocidental, Lisbon, PRT; 2 Internal Medicine, Hospital Professor Doutor Fernando Fonseca, Amadora, PRT

**Keywords:** atopic, bronchiolitis, respiratory tract infections, prevention, hospitalization, severe exacerbation, wheezing

## Abstract

Introduction

Wheezing is common in preschool-aged children, affecting about half of all children within their first six years of life. Children who have recurrent wheezing experience disease-related morbidity, including increased emergency visits and hospitalizations. Early-life lower respiratory tract viral infections are linked to recurrent wheezing and eventual asthma onset. Identifying high-risk children is crucial, with the frequency and severity of wheezing episodes being good predictors of long-term outcomes.

Aim

To identify predictors of severe exacerbations in children with recurrent wheezing.

Methods

We conducted a retrospective cohort study involving 168 pediatric patients with recurrent wheezing followed up at our outpatient clinic. The outcome of interest was the occurrence of a severe exacerbation, defined as any exacerbation requiring hospitalization and the need for supplemental oxygenation or ventilatory support.

Results

The median age of the first wheezing exacerbation was five months, with a predominance of the male gender. Approximately two-thirds of the patients had a family history of atopy. Comorbid allergic rhinitis and atopic dermatitis were present in 15.4% and 16.7% of patients, respectively. Twenty percent of patients had a severe wheezing exacerbation as the first form of presentation, and 30% presented at least one severe exacerbation from the first presentation to the last follow-up. Patients with severe exacerbations were younger at the first episode (median age 4 months, IQR 2-7, versus 7 months, IQR 4-12, p=0.027) and more frequently had a family history of atopy (71.7% versus 55.6%, p=0.050). In this cohort, patients who initially presented with a severe episode are at increased risk of incident severe exacerbations during follow-up, HR 2.24 (95%CI 1.01-4.95).

Conclusions

We know that the severity of exacerbations in children with recurrent wheezing correlates with the long-term outcomes of the disease. Therefore, preventing severe exacerbations can positively impact the prognosis of these patients. In this analysis, we found independent predictors of severe exacerbations to be the first clinical episode before the age of three months and a family history of atopy. We also found that patients whose initial presentation was severe have a higher risk of new severe exacerbations. Therefore, these subgroups of patients should be closely monitored by pediatricians.

## Introduction

Wheezing is very prevalent during the preschool years, affecting approximately half of all children within their first six years of life [1,2]. Among those who have episodes of wheezing before reaching the age of three, around 40% continue to experience it by the time they turn six [3,4]. Preschool children with recurrent wheezing (RW) encounter disease-related morbidity, with nearly double the rate of emergency department visits and an almost threefold higher rate of hospitalization compared to older children [3,4].

The presence of wheezing implies a limitation of expiratory flow, but the symptom does not provide any clue about the underlying cause. It is more commonly associated with lower respiratory tract viral infections (LRTVI) in young children, typically respiratory syncytial virus (RSV) and rhinovirus. Infection-induced wheezing may first manifest as acute bronchiolitis, a frequent antecedent to recurrent infection-induced wheezing [3]. Besides their role in triggering acute episodes of wheezing, LRTVI early in life have been linked with RW and the eventual onset of asthma [3].

Prompt identification of children at high risk of continuing to wheeze in school age is essential, having clinical and prognostic implications. The frequency and severity of episodes serve as better predictors of long-term outcomes [5]. Many patients with severe episodes of virus-induced wheezing continue to present symptoms until 5-10 years of age, with some experiencing symptoms between episodes [5]. Phenotypic characterization has many limitations in predicting high-risk children since there is significant overlap between phenotypes, and the symptom pattern varies over time [4,5].

On the other hand, the efficacy of childhood asthma prediction models in clinical application remains uncertain. A systematic review demonstrated that none of the reviewed models combined good performance in both predicting and excluding asthma development simultaneously [6,7]. Some of the well-known risk factors include family history of asthma or allergy, exposure to tobacco, comorbid atopic disease (allergic rhinitis or atopic dermatitis), evidence of allergic sensitization, severity of early asthma-like symptoms, and frequent respiratory infections, especially at an early age. Quantitative assessment of atopy through measurements of eosinophilia and immunoglobulin E (IgE) can also be useful [6-8].

Our aim was to identify predictors of severe exacerbations with the goal of implementing strategies for their prevention.

## Materials and methods

Patients

This was a retrospective single-center cohort study. All patients with RW who are routinely followed up at our Pediatric Allergy and Pneumology outpatient clinic and had their first consultation between January 2016 and December 2021 were included. For the definition of RW, we based our criteria on the 2023 Global Initiative for Asthma. Thus, we included children who experienced at least three episodes per year of wheezing, with or without symptoms between the episodes [9].

Demographic, clinical, laboratory, and imaging data were retrieved from patients' clinical charts and collected into a specific anonymized database. Patients with missing information regarding clinical presentation or follow-up data were excluded from the analysis. This retrospective study followed the regulations of the Local Research Ethics Committee. All procedures were performed in accordance with the ethical principles expressed in the 2013 Declaration of Helsinki.

Variables and outcomes

The outcome of interest in this study was the occurrence of a severe exacerbation of RW, defined as any exacerbation requiring hospitalization for at least a 24-hour period and the need for any degree of supplemental oxygenation or ventilatory support.

A family history of asthma was considered when there was at least one first-degree relative with asthma, and a family history of atopy was defined as having at least one first-degree relative with asthma and/or another atopic disease other than asthma. Air allergen sensitization was assessed with skin-test reactivity and allergen-specific IgE levels. Children with inter-crisis symptoms were those who exhibited wheezing, cough, or shortness of breath during sleep, play, or when laughing. Maintenance treatment during follow-up was defined as at least three months of treatment with daily inhaled corticosteroids and/or a leukotriene receptor antagonist. The diagnosis of asthma was made in children with a history of respiratory symptoms, such as wheezing, shortness of breath, and cough, that vary over time and in intensity, with family and/or personal history of atopy, and who exhibit a good response to bronchodilator treatment during exacerbations and to maintenance treatment. The follow-up time considered was from the first clinical episode of wheezing until the last hospitalization or consultation at our outpatient clinic.

Statistical analysis

Analysis was performed using STATA MP version 14 (StataCorp LLC®, Texas). Categorical data were presented as frequency counts and percentages, and analyzed with the chi-square test and Fisher’s exact test, as appropriate. Normality was tested with the Kolmogorov-Smirnov test. Skewed distributions were described with medians and interquartile ranges (IQR) and compared with the Mann-Whitney test. Normal distributions were described with means and standard deviations and compared with Student's t-test. The two-sided alpha level was set at 0.05. The 95% confidence intervals (95%CI) were shown. For outcome measurements, analyses were separately performed. To assess the risk of occurrence of any severe exacerbation, demographic, clinical, laboratory, and treatment variables were selected for logistic regression analysis, assessed by the estimated odds ratio (OR) with 95%CI. Variables yielding p-values of less than or equal to 0.10 in the univariate analyses were then entered into a multiple logistic regression model. To assess the incidence of severe exacerbation during follow-up, after the first episode of wheezing, a survival analysis using the Kaplan-Meier method was used, and the estimated hazards ratio (HR) was calculated accordingly.

## Results

A total of 168 pediatric patients with RW were included in this study. The median age of the first wheezing exacerbation was five months (IQR 2-11) and 61 patients (36.3%) were female. The general characteristics of the study population are summarized in Table 1. A history of prematurity was present in 26 patients (15.5%), and bronchopulmonary dysplasia in only three (1.9%). A family history of atopy and asthma in first-degree relatives was present in 60.7% and 46.4% of patients, respectively. Some patients had comorbid atopic diseases (15.4% with allergic rhinitis and 16.7% with atopic dermatitis). A history of smoking in cohabitants was described in 34.5% of cases. A total of 25 patients (14.9%) developed sensitization to air allergens during follow-up, and 30 (23.2%) had symptoms between wheezing exacerbations, most commonly cough. Thirty-four (20.2%) of the patients had a severe wheezing exacerbation requiring hospitalization as the first form of presentation. In total, 53 (30.4%) presented with at least one severe exacerbation from the first presentation to the last follow-up; the median number of severe exacerbations was 1 (1-2). Maintenance treatment was implemented in 138 patients (82.1%).

**Table 1:** Demographic characteristics of the study population IQR: interquartile range

Characteristics	n=168
Female sex, n (%)	61 (36.3%)
Age of first event in months, median (IQR)	5 (3-11)
Prematurity, n (%)	26 (15.5%)
Bronchopulmonary dysplasia, n (%)	3 (1.9%)
Family history	
Asthma, n (%)	78 (46.4%)
Atopy, n (%)	102 (60.7%)
Allergic rhinitis, n (%)	26 (15.4%)
Atopic dermatitis, n (%)	28 (16.7%)
Cohabitants smoking, n (%)	58 (34.5%)
Frequents nursery, n (%)	150 (89.3%)
Sensitization to air allergens, n (%)	25 (14.9%)
Number of wheezing episodes per year, median (IQR)	4 (3-5)
Presence of inter-crisis symptoms, n (%)	30 (23.2%)
Maintenance treatment during follow-up, n (%)	138 (82.1%)
First exacerbation was severe, n (%)	34 (20.2%)
Any severe exacerbation, n (%)	53 (30.4%)
Diagnosis of asthma during follow-up, n (%)	6 (3.6%)
Time of follow-up in months, median (IQR)	27 (13-43)

Table 2 depicts the comparison between patients presenting with at least one severe exacerbation and those with no severe exacerbations during the entire follow-up. Patients with severe exacerbations were younger at the first episode, with a median age of four months (2-7) versus seven months (4-12) (p=0.027). Compared to patients with no hospitalizations, patients with severe wheezing attacks also had more frequently a family history of atopy (71.7% versus 55.6%, p=0.050). Severe exacerbations seemed to occur less frequently in patients with a personal history of allergic rhinitis and sensitization to air allergens (Table 2). Patients with severe exacerbations were more likely to receive inhaled corticosteroids as maintenance treatment (90.6% versus 78.9%, p=0.072). In the multivariate logistic regression model, the independent predictors of severe exacerbations were an age of first presentation of less than three months, OR 3.48 (95%CI 1.67-7.53), and a family history of atopy in a first-degree relative, OR 2.36 (95%CI 1.09-5.09).

**Table 2:** Comparison of patients with and without severe exacerbations and logistic regression analysis IQR: interquartile range, OR: odds ratio, CI: confidence interval

Variable	Severe, n=53	Not severe, n=115	p-value	OR	95%CI	p-value
Female sex, n (%)	16 (27.1%)	43 (37.4%)	0.408			
Age of first event in months, median (IQR)	4 (2-7)	7 (4-12)	0.027			
Age of first event < 3 months, n (%)	23 (43.4%)	21 (18.3%)	0.001	3.48	1.61-7.53	0.002
Age of first event < 6 months, n (%)	37 (69.8%)	35 (47.8%)	0.009			
Prematurity, n (%)	10 (18.9%)	16 (13.9%)	0.411			
Family history						
Asthma, n (%)	26 (49.1%)	52 (45.2%)	0.643			
Atopy, n (%)	38 (71.7%)	64 (55.6%)	0.050	2.36	1.09-5.09	0.028
Allergic rhinitis, n (%)	4 (7.6%)	22 (19.1%)	0.063	0.51	0.15-1.78	0.292
Atopic dermatitis, n (%)	9 (17.0%)	19 (16.5%)	0.941			
Cohabitants smoking, n (%)	16 (30.2%)	42 (36.5%)	0.423			
Frequents nursery, n (%)	50 (94.3%)	100 (86.9%)	0.162			
Sensitization to air allergens, n (%)	4 (7.7%)	21 (18.3%)	0.086	0.56	0.16-1.95	0.363
Number of wheezing episodes per year, median (IQR)	3 (2-4)	3 (2-4)	0.854			
Presence of inter-crisis symptoms, n (%)	6 (15.1%)	92 (19.1%)	0.527			
Maintenance treatment during follow-up, n (%)	48 (90.6%)	90 (78.9%)	0.072	2.03	0.69-5.95	0.197

**Figure 1:**
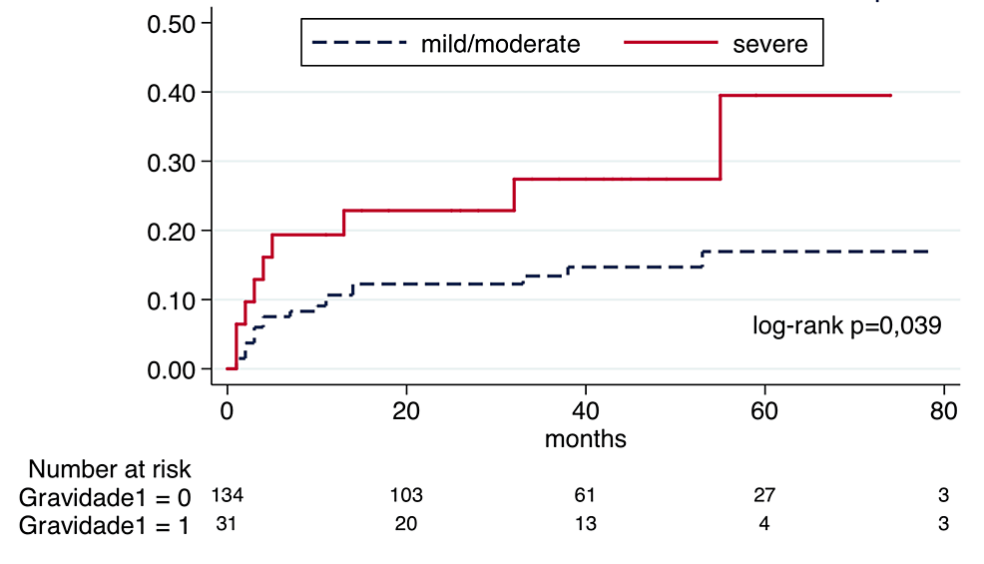
Incidence of severe exacerbations after the first episode

Then, the incidence of severe exacerbations after the first episode of wheezing was assessed. For this analysis, the first episodes of wheezing that were severe (the initial presentation in 20.2% of patients) were not considered, as we intended to assess only incidental severe exacerbations after the initial episode. Figure 1 shows that patients who initially presented with a severe episode are at an increased risk of incident severe exacerbations during follow-up, with an HR of 2.24 (95%CI 1.01-4.95). All other variables included in this study did not show significance in the survival analysis, including the age at the first episode (p=0.748) and maintenance treatment (p=0.176).

## Discussion

In our sample, nearly one-third of the patients experienced at least one severe exacerbation during follow-up. Hence, it is important to identify reliable predictors that would enable us to implement strategies for preventing severe exacerbations, which are still poorly defined in this age group [10]. Our study revealed that children experiencing severe exacerbations during follow-up had their first wheezing episode at a younger age and were more likely to have a family history of atopy. On the other hand, a personal history of allergic rhinitis and evidence of sensitization to aeroallergens were not predictors of exacerbation severity. This may be explained by the early age of the patients during severe exacerbations, preceding allergenic sensitization, and the diagnosis of rhinitis. In fact, early wheezing is not correlated with atopic dermatitis or allergic rhinitis in school-age children and adolescents [11].

Our analysis also showed that patients initially presenting with a severe exacerbation are at a higher risk of experiencing subsequent severe episodes. Indeed, some authors argue that the first episode of severe bronchiolitis or wheezing before the age of two is a critical event and an opportunity to initiate asthma prevention strategies [12]. The association between early severe bronchiolitis, RW, and asthma is well established, but a definitive causality mechanism and the complete pathophysiology are yet to be fully understood. Several studies have demonstrated that hospitalization due to RSV infection is associated with more severe forms of asthma [13]. This relationship involves aberrant immunological pathways like Th2/Th17-dominant response, especially in the setting of atopy, genetic predisposition, and environmental exposures [13]. Genetic susceptibility to asthma has early phenotypic effects, influencing the wheezing phenotype from the first months of life and the development of asthma in school-age children, justifying the importance of adopting preventive strategies [14,15]. The literature suggests that the daily administration of inhaled corticosteroids has shown a reduction in exacerbations in preschool children, particularly in those with persistent symptoms [10]. However, in our study, there was no significant relationship between maintenance treatment and the propensity to have severe exacerbations.

Limitations of this study include its retrospective nature and the fact it was a single-center cohort analysis. In fact, the results of this work should be validated in prospective cohort studies. Nevertheless, this was a well-characterized cohort reflecting uniform management by the same physicians, with homogeneous clinical protocols, which are strengths of our study. It should also be noted that it is uncertain whether these results could be applied to patients with wheezing who do not fulfill the clinical criteria for RW, as these patients were not included in this analysis. This issue should be addressed in future studies. Finally, the follow-up time for this analysis was not sufficient to assess the risk for the development of asthma in many patients who were still young at the last follow-up, for which a longer period of analysis should be warranted to address this objective.

## Conclusions

In summary, in the cohort of pediatric patients with RW, a first clinical episode before the age of three months and a family history of atopy were independent predictors of severe exacerbations. Moreover, patients who presented with a first severe episode were at increased risk of new severe exacerbations. Therefore, these subgroups of patients should be closely monitored by pediatricians.
